# Role of diet and its effects on the gut microbiome in the pathophysiology of mental disorders

**DOI:** 10.1038/s41398-022-01922-0

**Published:** 2022-04-20

**Authors:** J. Horn, D. E. Mayer, S. Chen, E. A. Mayer

**Affiliations:** 1 G. Oppenheimer Center for Neurobiology of Stress and Resilience, Vatche and Tamar Manoukian Division of Digestive Diseases, David Geffen School of Medicine at UCLA, Los Angeles, CA USA; 2 MayerInterconnected, LLC, Los Angeles, CA USA; 3 University of California, San Francisco, CA USA

**Keywords:** Depression, Human behaviour, Autism spectrum disorders, Molecular neuroscience

## Abstract

There is emerging evidence that diet has a major modulatory influence on brain-gut-microbiome (BGM) interactions with important implications for brain health, and for several brain disorders. The BGM system is made up of neuroendocrine, neural, and immune communication channels which establish a network of bidirectional interactions between the brain, the gut and its microbiome. Diet not only plays a crucial role in shaping the gut microbiome, but it can modulate structure and function of the brain through these communication channels. In this review, we summarize the evidence available from preclinical and clinical studies on the influence of dietary habits and interventions on a selected group of psychiatric and neurologic disorders including depression, cognitive decline, Parkinson’s disease, autism spectrum disorder and epilepsy. We will particularly address the role of diet-induced microbiome changes which have been implicated in these effects, and some of which are shared between different brain disorders. While the majority of these findings have been demonstrated in preclinical and in cross-sectional, epidemiological studies, to date there is insufficient evidence from mechanistic human studies to make conclusions about causality between a specific diet and microbially mediated brain function. Many of the dietary benefits on microbiome and brain health have been attributed to anti-inflammatory effects mediated by the microbial metabolites of dietary fiber and polyphenols. The new attention given to dietary factors in brain disorders has the potential to improve treatment outcomes with currently available pharmacological and non-pharmacological therapies.

## Introduction

Psychiatric disorders have traditionally been considered diseases of the brain, with little role of the body or individual organs in their pathophysiology. Exceptions to this brain-focused approach have been pre-scientific concepts in Traditional Chinese Medicine, Ayurvedic Medicine, and Hippocratic Medicine, all of which attributed a significant role of the body, in particular the digestive system and diet, in modulating mental processes. Modern psychosomatic medicine has posited that stress, emotional, and cognitive factors can influence body functions.

Early evidence suggesting a role of altered gut to brain signaling in anxiety, depression, and autism spectrum disorder (ASD) have come from clinical anecdotal observations in patients with these diagnoses and associated GI manifestations. In many of these studies, psychiatric conditions were viewed as co-morbid conditions to the primary diagnosis of a gut disorder. In addition, a number of large epidemiological studies have implicated dietary factors in some of these disorders [1–5], both in terms of risk factors [6] as well as potential therapies [2, 5, 7]. However, none of these studies have been able to establish a causative role of the gut or dietary factors in psychiatric disease to date.

### Diet can affect the brain via multiple mechanisms

The importance of sufficient macro- and micronutrients for normal brain development and the role of nutrient deficiencies early in life for compromised brain function have long been known [8, 9]. While the great majority of studies to date has focused on dietary components such as amino acids and micronutrients that are completely absorbed in the proximal small intestine, there has been a growing interest in food molecules that are too large to be absorbed intact in the proximal gut, and whose absorption largely relies on metabolism by the gut microbiota in the distal small intestine and colon. The health benefit of these non-absorbable dietary components is crucially dependent on the composition and functions of the gut microbiome.

The exponential progress in microbiome science following the Human Microbiome Project [10] and some of the paradigm challenging results from early rodent studies about the influence of the gut microbiome on emotion-like behavior and brain biochemistry have introduced the concept of the BGM axis (or better BGM system) playing a role in many psychiatric disorders [11–13]. While these pioneering studies had a major influence on our understanding of the role of gut microbes in mammalian behavior, few of their findings have been translatable into the diagnosis or treatment of human psychiatric disorders to [12]. However, as diet has a major influence on human gut microbial composition and function, the notion that diet in addition to direct effect of macro and micronutrients on the brain could play a causative role in gut microbiome alterations with impacts on human emotional and cognitive function, has become an exciting research topic in psychiatry, and the term Nutritional Psychiatry has been proposed [11, 14, 15].

Nutritional psychiatry is a relatively new field of research that has developed from revolutionary preclinical observations and a series of large, cross-sectional, epidemiological studies, linking diet with different aspects of mental health, and from the insights gained from microbiome science which has provided a link between diet, microbial function, and brain health. Converging results from these studies support a potential role of diet, and a possible beneficial role of particular dietary interventions in different brain disorders, including, but not limited to depression, cognitive decline, and Alzheimer’s disease (AD), ASD, and certain forms of epilepsy (for a complete list of such disorders, see Table 1). A growing number of interventional and mechanistic studies have confirmed a beneficial effect of a mostly plant-based diet, high in fiber and polyphenols, on mental health.

**Table 1:** Evidence for the effect of dietary interventions on brain disorders.

Disorder	Dietary intervention therapies
**Depression**	Epidemiological, interventional studies, and meta-analysis of RCTs revealed that intervention with a largely plant-based diet can reduce depressive symptoms compared to control conditions (Jacka et al., 2017; Parletta et al., 2019; Sanchez-Villegas et al., 2013). A large population-based study found a positive correlation between *Coprococcus* and *Dialister* with quality of life, and a depletion of these taxa in treatment-free depression. Participants with low relative abundance of *Bacteroides* showed lower quality of life scores and higher prevalence of depression (Valles-Colomer et al., 2019).
**Anxiety**	Meta-analysis of 11 RCTs from 2270 individuals showed no overall effect of dietary interventions on anxiety compared with control conditions (g = 0.100, 95% CI = − 0.036 to 0.235, p = .148, Q = 18.5, I2 = 46.1). As with depression outcomes, some studies using mostly (>75%) female participants observed significant positive effects on anxiety from dietary interventions (*n* = 6, *n* = 965, g = 0.211, 95% CI = 0.09 to 0.34, *p* = 0.001), whereas those with predominantly male participants observed non-significant negative effects (g = −0.19, 95% CI = − 0.42 to 0.04, *p* = 0.107) (Firth et al., 2019).
**Parkinson’s Disease**	The MIND (Mediterranean-DASH Intervention for Neurodegenerative Delay) diet consisting of higher consumption of berries and green leafy vegetables than the traditional Mediterranean diet resulted in a significantly lower risk for parkinsonism as well as a slower rate of PD symptom progression relative to controls in a study with 706 participants of an age range between 59 and 97. The group on a Mediterranean diet showed a significant reduction in parkinsonism progression when compared to the control group (Agarwal et al., 2018).
**Alzheimer’s Disease**	Early-stage clinical studies show positive causal evidence for a ketogenic diet to improve cognitive function in those with AD despite the heterogeneity of interventional dietary studies. However, there is a paucity of evidence supporting an effect of a ketogenic diet on the prevention of AD development, an area of potential future research (Krikorian et al., 2012; Henderson et al., 2009, Ota et al., 2019; Reger et al., 2004; Taylor et al., 2018; Neth et al., 2020; Morrison et al., 2020; Fortier et al., 2019). The NUAGE dietary intervention trial showed that adherence to a Mediterranean diet was associated with increased abundance of butyrate producing taxa, which were negatively associated with inflammatory markers and positively associated with enhanced cognition (Ghosh et al., 2019). Supplementation with probiotics for 12 weeks induced a significant improvement in Mini- Mental State Examination score (Akbari et al., 2016).
**Autism Spectrum Disorder**	Various small, low quality dietary intervention studies have shown improvement in several domains compared to control groups, such as communication, social interaction, inattention, and hyperactivity (Cade et al., 2000; Knivsberg et al., 2002; Elder et al., 2006; Whiteley et al., 2010; Adams et al., 2018; Grimaldi et al., 2018). Metabolic and endocrine pathways have been observed to be different in ASD individuals compared to healthy controls (Needham et al., 2021; Emond et al., 2013). No strong causal evidence for diet-induced therapeutic microbiome changes. MTT was associated with a significant sustained decrease in GI symptoms and ASD symptoms, and favorable changes in the abundance of certain beneficial bacterial taxa (Kang et al., 2017; Kang et al., 2019).
**Epilepsy**	Meta-analysis of 10 RCTs found weak positive evidence of seizure reduction of the dietary intervention groups relative to the control groups (McGill et al., 2018). A case-control study demonstrated a 50% reduction in seizures in children with DRE after one week of being on the ketogenic diet, associated with decreased levels of several microbial taxa (Xie et al., 2017). Another ketogenic dietary intervention study showed no significant change in alpha diversity but diminished relative abundance of the butyrate producing taxa *bifidobacteria*, *E. rectale*, and *Dialister* and increase of *E. coli* (Lindefelt et al., 2019). In children treated with a ketogenic diet for six months, a decrease of *Firmicutes* and *Actinobacteria* and increased levels of *Bacteroidetes* were observed. The subgroup with increased abundance of *Alistipes*, *Clostridiales*, *Lachnospiraceae*, *Ruminococcaceae*, and *Rikenellaceae* had a less than 50% reduction in seizures compared to other subgroups (Zhang et al., 2018).
**Eating Disorders**	High adherence to a Mediterranean diet in 11.1% of 1472 subjects at high risk for binge eating disorder was associated with decreased development of the disorder (Bertoli et al., 2015). A study with 11,800 women with either anorexia nervosa or bulimia nervosa showed evidence for a potential inverse association between a Mediterranean dietary pattern and both eating disorders (Leone et al., 2018). Significant differences in the relative abundance of certain gut microbiota have been observed in anorexia nervosa (Kleiman et al., 2015; Morita et al., 2015).
**ADHD**	In a study with 100 children randomly assigned to either the dietary or the control group, the ADHD rating scale score between baseline and the first phase of the dietary intervention was significantly lower in the group following a restricted elimination diet compared to the control group (Pelsser et al., 2017).The beta diversity of the gut microbiome of ADHD participants was different than in the control group, even though the changes of individual bacterial taxa were different (Aarts et al., 2017; Prehn-Kristenen et al., 2018).

In this review, we will first discuss the emerging science about the bidirectional communication within the BGM system, and then review the existing animal and human literature supporting a role for diet and supplements in influencing the brain, psychiatric pathophysiology, and symptoms. We will focus on a limited and non-exhaustive number of mechanisms which have been implicated in several brain disorders, and which illustrate different ways by which diet-related gut microbial molecules, metabolites and mechanisms can affect the brain, in particular short chain fatty acids, tryptophan (Trp) metabolites, bile acid (BA) metabolites, and immune-mediated processes. From the large number of psychiatric disorders which have been associated with diet and the microbiome, we have limited the discussion to those disorders for which sufficient scientific evidence from preclinical and clinical studies is available to suggest a causative role. We will point out the paucity of well controlled longitudinal, interventional clinical studies (RCTs), which identify a causality between a specific diet, or supplements, and a psychiatric disorder. We will also discuss potential future implications of Nutritional Psychiatry, such as the proposed role of diagnostic testing of the gut microbiome to identify targets for personalized treatments and will discuss the potential for integrative approaches combining dietary interventions, pharmacotherapy, and cognitive behavioral approaches.

### The brain gut microbiome system

**Fig. 1:**
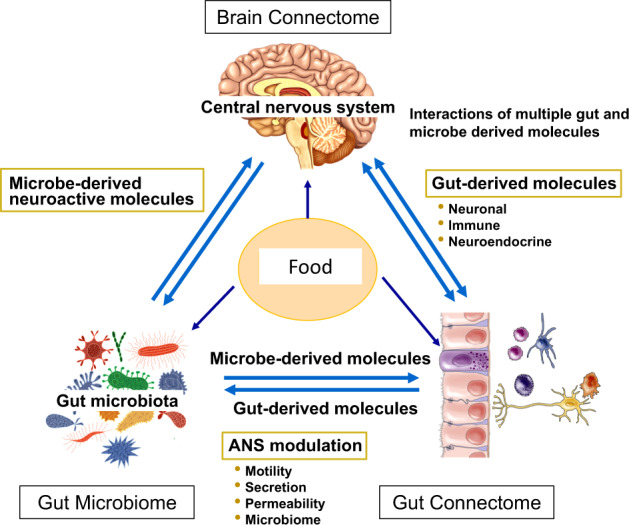
The influence of food on the brain gut microbiome system. The brain connectome, gut connectome and microbiome make up the 3 hubs in the larger BGM network. All hubs are linked by bidirectional connections with multiple feedback loops generating a non-linear system. Different components of food influence the brain, the gut and the gut microbiome via different communication channels. Dietary components can influence the gut directly and reach the brain after absorption in the small intestine. In addition, diet can influence gut microbial composition and diversity, and after microbial metabolism can modulate the gut connectome. Some of the microbial derived molecules are absorbed and reach the brain via the systemic circulation and/or the vagus nerve (see Fig. 2) Similarly, the brain can modulate the microbiome directly through the effect of neuroactive substances released into the gut lumen affecting gene expression and behavior of microbes, or indirectly via alterations of the gut microbial environment. Modified with permission from Martin et al., 2018.

Emerging evidence supports a model of bi-directional communication between the central nervous system (CNS), the gut, and its microbiome, collectively referred to as the BGM system (Fig. 1). As discussed throughout this review, a number of dietary effects on the brain are mediated by the BGM system, and a general knowledge of this system is required to better understand many aspects of dietary modulation of the brain. The gut microbiome has been shown to interact with the brain primarily through three interacting pathways, namely neuronal, endocrine, and immunoregulatory [12, 16]. In turn, the CNS can directly influence the composition and function of the gut microbiota through the autonomic nervous system [17]. This top-down modulation can occur indirectly via regulation of gastrointestinal (GI) motility and transit, mucus secretion and permeability of the intestinal barrier, and luminal release of neurotransmitters. In addition, direct modulation of gut microbial gene expression and function by norepinephrine and likely other neurotransmitters released from postsynaptic sympathetic terminals has been reported [18]. Evidence for a similar release of stress-induced serotonin from enterochromaffin cells into the gut lumen has been reported, with some microbes exhibiting a serotonin transporter in their cell membranes [19]. The functional consequences of this luminally released serotonin (as well as other signaling molecules stored in specialized gut cells) remains to be determined.

### Neuroendocrine communication channel

Many microbes produce metabolites from dietary components (complex carbohydrates, amino acids), bodily secretions (BAs, estrogens), or chemical substances, so called xenobiotics (including pesticides and some medications). Many of these metabolites have been shown to influence brain structure and function in preclinical studies [20, 21].

Gut microbes communicate with a variety of cells of the gastrointestinal endocrine system [22]. Enteroendocrine cells (EECs) are interspersed in the gut epithelium and contain important signaling molecules, including key orexigenic (ghrelin) and anorexigenic (NPY, PYY) hormones which can act locally on the vagus nerve as neurotransmitters, or reach the CNS via the systemic circulation in an endocrine fashion [12]. The interaction of such hormones in the periphery and in the hypothalamus play a key role in the regulation of appetite and satiety [23] and a dysregulation of these signaling systems has been implicated in obesity and food addiction [24]. Enteroendocrine and enterochromaffin cells (ECCs) form close synaptic connections with certain vagal afferent fibers through cell extensions called neuropods [25, 26]. While these gut hormones are also released into the systemic circulation and reach the brain directly, these synaptic connections function in the rapid relay of a nutrient and other signals from the gut to the brain.

**Fig. 2:**
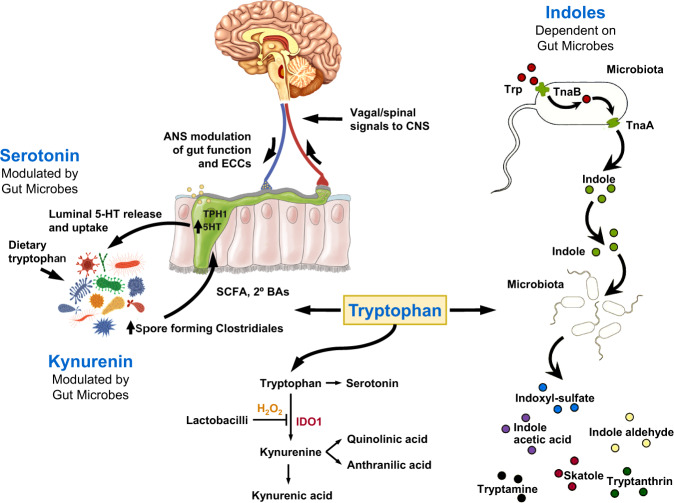
Gut microbes generate neuroactive metabolites from tryptophan. The essential amino acid Tryptophan is the precursor for a number of neuroactive signaling molecules including serotonin, kynurenine and indoles. Whereas microbes only play a *modulatory* role in the generation of serotonin and kynurenine, the synthesis of indoles is fully dependent on gut microbial metabolism. The relative abundance of the 3 metabolites is dependent on tryptophan intake, on the relative abundance of involved microbial taxa and on stress induced input from the autonomic nervous system. Modified with permission from Martin et al., 2018.

The essential amino acid Trp is a precursor to serotonin, as well as to other important metabolites in neuroendocrine signaling (Fig. 2). Specific gut microbiota play a critical role in the modulation of Trp into various metabolites, which include but are not limited to kynurenine, indoles, and tryptamine [19, 27, 28]. Trp metabolites are important contributors to neuroendocrine and neuroimmune mechanisms as they can act on the CNS either through the bloodstream or via vagal afferent signaling [18].

The great majority of the body’s serotonin (95%) is produced and stored in ECCs and plays an important role in modulating the activity of the enteric nervous system and in signaling to the brain via different subtypes of vagal afferents which form synaptic contacts with ECCs [29]. Microbial metabolites (SCFAs and BAs) have been shown to stimulate the production and release of serotonin by ECCs [19]. By regulating the serotonergic system, gut microbes can directly influence their environment [18]. While serotonergic neurons located in the brainstem show widespread projections to the brain and are well-known to play an important role in modulating vital functions such as sleep, food intake, mood regulation and pain, gut-based serotonin plays an important role in gastrointestinal motility and secretion [12]. Germ-free mice have been demonstrated to have half the amount of serotonin when compared to mice with a normal gut microbiome [28].

Another Trp metabolite is kynurenine, the synthesis of which is modulated by *Lactobacillus* taxa (Marin et al., 2017). *Lactobacilli* produce hydrogen peroxide, a reactive oxygen species which normally suppress host kynurenine metabolism by inhibiting the expression of the enzyme indoleamine-2,3-dioxygenase (IDO1). IDO1 plays part in the synthesis of kynurenine from Trp in the GI tract (Schwarcz et al., 2012). In a rodent model of chronic variable stress, the stress-induced reduction of *Lactobacillus* decreased hydrogen peroxide-mediated inhibition of IDO1, resulting in an increased synthesis of kynurenine from Trp, (Valladares et al., 2013, Vujkovic-Cvijin, 2015). In these studies, higher kynurenine concentrations in the brain were correlated with increased depression-like behavior which was reduced by the administration of *Lactobacillus* [30]. Kynurenine, which can pass the blood-brain barrier, has been shown to produce neuroinflammation and neurodegeneration, which have also been reported in AD and depression [31].

A third group of Trp metabolites are indoles, which are solely produced by gut microbes. Indoles are precursor molecules to many compounds that are critical for brain health and function and have been detected in the GI tract, brain, and the systemic circulation [32]. While many indoles are known to positively influence both systemic and intestinal homeostasis [20], preclinical studies have demonstrated that some indole metabolites may also exert negative effects on brain health, such as causing depression-like behavior [33]. While gut microbes play only a modulatory role in both serotonin and kynurenine production from Trp, the indole pathway is completely microbe dependent, as only certain microbes possess the enzyme tryptophanase required for their production from Trp [34]. Indoles are further metabolized in the liver and are precursor molecules to many compounds that are critical for brain health and function. They have been detected in the GI tract, brain, and the systemic circulation [32]. One such metabolite, indoxyl sulfate, may play part in the pathophysiology of several brain disorders, including ASD, AD, and depression [12].

In summary, the interactions of gut microbes with dietary tryptophan leading to the generation of multiple neuroactive metabolites, some of which have been implicated in several brain disorders, clearly shows the intricate connection between diet, the gut and certain gut microbes, and brain diseases.

### The immune communication channel

**Fig. 3:**
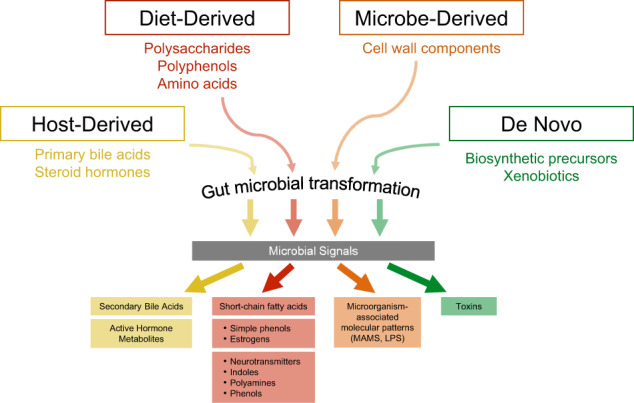
Four sources for gut microbial signaling molecules. Gut microbial signaling molecules are derived from at least 4 different sources: Diet-derived, microbe-derived, host-derived and newly synthesized molecules. Chemical transformation of these molecules results in a vast number of signaling molecules which can influence not only cells in the gut (immune, nerve, endocrine cells), but following dissemination throughout the body are able to modulate all organs, including the brain. Certain diet-derived microbial metabolites have neuroactive effects on the central and autonomic nervous system, while microbial cell wall components can activate the immune system by interacting with TLRs. Some microbial metabolites (in particular the SCFA butyrate) exert anti-inflammatory effects. Modified with permission from Needham et al., 2020.

Lipopolysaccharides (LPS) and microbe associated molecular patterns (MAMPs) contained in the cell wall of gram-negative microbes can interact locally with receptors on enteric neurons or vagal afferents (so called toll-like receptors or TLRs), with cells of the gut-based immune system, or exert their effects distally throughout the body, including the brain. Many studies have investigated the complex interactions between the gut microbiota and CNS inflammation. The gut microbiome can influence central immune activation through direct activation of the gut based immune system and trigger pro- and anti-inflammatory systemic immune responses [35]. The involvement of neuroinflammatory and neurodegenerative mechanisms related to the BGM system which may play a role in various brain disorders has recently been reviewed (Fig. 3) [13].

While cell wall components of gut microbes play an important role in neuroinflammation, other microbes play an important role in preventing and counteracting immune activation in the gut. *Ackermansia* strains are involved in the regulation of the intestinal mucus layer, an important component of the gut barrier. Another group of microbial metabolites, in particular short chain fatty acids (SCFAs) exert powerful anti-inflammatory effects. SCFAs, in particular butyrate, are produced by a limited number of gut microbes through fermentation of complex carbohydrates, such as resistant starch. This group of beneficial microbes include the taxa *Faecalibacterium prausnitzii* (*F. prausnitzii), Eubacterium rectale (E. rectale), E. hallii*, and *Ruminococcus bromii (R. bromii*) [36]. They mediate their anti-inflammatory effects (Topping et al. 2001; Russell et al. 2011) by promoting TH1 cell mediated IL-10 production via G-protein coupled receptors 43 (GPR43). In addition, butyrate has also been shown to downregulate the expression of genes involved in inflammatory pathways in non-inflamed intestinal tissue [37].

There is a close link between immune activation in the gut and neuroinflammation in the brain, as the gut microbiome can directly influence the maturation and functioning of microglia in the CNS as part of the immunoregulatory pathways. Microglia make up the majority of immune cells in the brain, and gut bacteria play an important role in their proper functioning. In a rodent study, germ-free mice had a decreased number of mature microglia in various brain areas when compared to mice with a normal microbiome [38]. Another study with germ-free mice found functional defects in microglia and as a result observed heightened levels of proinflammatory cytokines IL-1B, IL-6, and TNF-alpha [39], indicating an impairment in immune function. Interestingly, impairments in microglia function were reversed by administration of SCFAs [38]. Dysregulation of microglia and gut microbial dysbiosis have been implicated in several psychiatric disorders, such as anxiety, depression, neurodegenerative disorders, such as Parkinson’s and multiple sclerosis, and neurodevelopmental disorders, such as ASD.

In summary, the close link between inflammatory signals originating in the gut in response to certain diets, in particular the Standard American Diet, and the crucial role of the gut microbiota in the generation of both pro- and anti-inflammatory signals emphasize the important, but incompletely understood interactions between diet, the gut microbiome, and brain diseases.

### Diet and brain health

In view of the key role that diet plays in the composition, richness, and function of the gut microbiome, and in view of the existence of multiple close communication between the microbiome and the brain via a network of interacting gut brain communication channels, it has become clear that the interactions of diet and the microbiota plays a crucial role in brain structure and function throughout the lifespan in multiple ways. While early (“first thousand days”) effects of nutrition on the developing brain have long been known, chronic dietary influences on the adult and aging brain have received increasing attention, in the context of cognitive decline, AD and Parkinson’s disease. Alterations in this influence on the brain as a result of unhealthy diets, or the opportunity to use gut microbiome targeted dietary interventions as a therapeutic approach have become the focus of Nutritional Psychiatry. There are several ways in which diet promotes healthy brain structure and function, which include reduction and prevention of metabolic endotoxemia, neuroactive metabolites, and essential micronutrients.

### Diet and metabolic endotoxemia

A number of preclinical and clinical studies have demonstrated a link between the Standard American diet (SAD), and an increase in markers of systemic immune activation, a phenomenon referred to as metabolic endotoxemia [37, 40], As reviewed elsewhere [12], metabolic endotoxemia results from a compromised gut barrier (“leaky gut”), allowing contacts between gut microbial cell wall components and Toll-like receptors on dendritic cells, or after translocation of intact microbes and activation of other cells in the gut-associated immune system. An increase in microbial taxa with anti-inflammatory function, such as *Faecalibacterium prausnitzii* (*F. prausnitzii), Eubacterium rectale (E. rectale), E. hallii*, and *Ruminococcus bromii (R. bromii*), contributes to a reduction in metabolic endotoxemia [41]. Dietary factors that increase the relative abundance of these anti-inflammatory and other health-promoting bacteria include prebiotics such as oligosaccharides and polyphenols some of which have prebiotic-like effects [42]. Prebiotics are defined as substrates which benefit host health because they are utilized by health-promoting microorganisms [43]. One of the main effect of prebiotics is to increase the relative abundance and activity of gut microbial taxa involved in the metabolism of complex carbohydrates into SCFAs [44]. Several clinical studies have shown the beneficial effects of a diet rich in prebiotics on the diversity and richness of the gut microbiome, and on the reduction of systemic immune activation [6, 45]. Such Mediterranean-like diets can promote healthy brain function as demonstrated in a variety of dietary intervention trials in depression and in cognitive decline [1–3, 6].

If supported by controlled longitudinal intervention studies, such a dietary approach combined with pharmacological and behavioral interventions holds significant promise as a new therapeutic comprehensive approach for psychiatric disorders, utilizing knowledge discovered in the novel field of Nutritional Psychiatry [14].

### Diet and neuroactive metabolites

A healthy diet may change the synthesis and secretion of neuroactive metabolites by gut microbes, which affects brain function and health. As discussed under neuroendocrine pathways of the last section, 95% of the body’s serotonin is produced in the gut by ECCs and enteric nerve cells with help of the microbiome [19]. The gut microbiome influences serotonin production and secretion as a direct result of the food we eat (in particular the amount of Trp), as diet modulates microbial composition [46]. A higher Trp concentration in the gut leads to increased serotonin synthesis in ECCs [18, 47]. In a rodent study, it was found that mice without an enteric microbiome had only half the amount of serotonin in the circulation when compared to mice with normal gut microbiota [48]. Stimulation of ECCs by microbes or intestinal contents moving through the gastrointestinal tract trigger the secretion of serotonin onto vagal sensory nerve endings and into the circulation and gut lumen [19]. This stimulation of the vagus nerve can result in signal relay to emotion-regulating brain areas [49]. In the CNS, serotonin produced and stored in the raphe nuclei as well as vagal signals generated by release of serotonin from ECCs is crucial to the regulation of sleep, appetite, pain sensitivity and mood [18].

### Diet and micronutrients

Specific micronutrients such as omega-3 fatty acids and zinc may have an important effect on brain development and function independent of the gut microbiome [50]. Nutritional research has moved away from focusing on single nutrients because they are never consumed in an isolated manner in real life [14]. Rather, a diet made up of a variety of different nutritious foods has been shown to have the most beneficial effect on both physical and mental health [11]. Nutrients derived from diet, such as vitamins, minerals, poly-unsaturated fats, and amino acids support healthy brain function [51–55]. Many of these nutrients serve as cofactors for enzymes, supporting for example the synthesis of neurotransmitters, myelination, cell signaling, and metabolic pathways [56]. A few specific nutrients have been intensely studied in their anti-depressant effect such as omega-3 fatty acids, folate, s-adenosyl-methionine, inositol, and vitamins B3, B6, and C and may be even beneficial if supplemented but only if adjunctive to a gut-healthy diet [50, 51, 57]. A high ratio of omega-6 to omega-3 fatty acids can contribute to a pro-inflammatory state due to the inflammatory properties of AA-produced eicosanoids and anti-inflammatory properties of EPA-produced eicosanoids [58]. Furthermore, low omega-3 fatty acid ingestion has been reflected in mental diseases such as depression [59].

In summary, a healthy diet rich in fiber, polyphenols and micronutrients has been shown to exert a positive effect on gut microbial composition, a reduction of metabolic endotoxemia and neuroinflammation, and has been associated with improvements in brain health. Fiber has been associated with improved brain health and function in a variety of small-scale observational and interventional studies [60]. Serotonin production and release in the enteric nervous system is greatly influenced by dietary choices as well, with the amounts of complex carbohydrates and tryptophan contained in the diet being the most important factors. Certain dietary micronutrients such as zinc, omega-3 fatty acids, folate, and B vitamins may further influence brain development and function, the lack of which can result in mental dysfunction and contribute to the development of brain disorders.

### Diet, the BGM system and brain disorders

Diet-related alterations in BGM system interactions have been implicated in several psychiatric and neurological disorders summarized in Table 1. In the following, we will discuss a limited number of these disorders for which significant clinical evidence supports such a relationship.

### Diet and depression

Depression (major depressive disorder, MDD, or clinical depression) has been increasing in prevalence, in particular in younger age groups [61]. Traditional therapies consist of centrally acting medications often in combination with cognitive behavioral strategies or other psychological strategies.

There have been a number of recent studies demonstrating that patients with MDD have an altered gut microbiome when compared to healthy controls [62, 63], although the nature of the dysbiosis in each of the studies varies, has not identified a consistent microbial biomarker, and causality between gut dysbiosis and clinical symptoms of depression has not been established. A recent large fecal microbiome population study using data from the Flemish Gut Flora Project investigated the correlation between gut microbiota composition, symptoms of depression, and indicators of quality of life. The study found a negative correlation between measures of physical functioning and the bacterial species *Flavonifractor*. The bacterial taxa *Coprococcus* and *Dialister* were found to be positively associated with quality of life and depleted in treatment-free depression. The participants with low relative abundance of *Bacteroides* showed lower quality of life scores and higher prevalence of depression. Furthermore, the GABA and Tryp metabolism pathways were enriched in gut health associated microbiota, indicating a potential role of gut microbes in these pathways [64].

Several studies have also shown that transferring the microbiome of a depressed individual into a healthy rodent (without or with suppressed gut microbiota) can induce depressive-like behaviors in the murine recipient, suggesting the possibility of a causal role for the microbiota in the pathophysiology of depression [63, 65]. In view of the important influence of diet on the gut microbiome, and the association of gut microbiome changes on depression-like behaviors, diet has emerged as a potential treatment strategy for depression (Table 1). A systematic review looked at twelve epidemiological studies to determine whether an association exists between diet quality and patterns in mental health in children and adolescents [66]. The study found evidence for a statistically significant relationship between unhealthy dietary patterns and poorer mental health, as well as for good-quality diet and better mental health in children and adolescents. However, in the cross-sectional studies included in this systematic review, no conclusions about causality of diet and mental health can be drawn, and the authors discussed several factors that could have influenced the results. One is the possibility that subjects with internalizing disorders or symptoms eat more unhealthy foods (“comfort foods”) as a form of self-medication. Another possibility is the influence of early eating habits and nutritional intake on brain development. And there is evidence that a nutrient-poor diet may lead to nutrient deficiencies, which have been associated with mental health issues [67, 68]. Consuming folate, zinc and magnesium is inversely associated with depressive disorders, whereas omega-3 fatty acids are inversely related to anxiety disorders.

Systemic immune activation has been implicated in the pathophysiology of depression [69]. Diets associated with metabolic endotoxemia, such as the SAD, may have a direct impact on various biological systems related to depression. Several studies that have reported strong correlations between mental well-being and reported happiness, and a healthy diet high in fresh fruits and vegetables [52–55]. Several reviews and meta-analyses have also backed this association. An analysis of four cohorts and nine cross-sectional studies showed that a reduced likelihood of depression was associated with a higher intake of fruit, vegetables, fish and whole grains [70]. A meta-analysis consisting of eight cohort studies and one case control, linked a reduced risk of depression with adherence to the Mediterranean diet [71].

One of the first intervention studies performed to date, the SMILES trial, involved a 12-week, parallel-group, single-blind randomized controlled trial (RCT) in 33 female and male participants with moderate to severe depression. Participants were randomized to receive either dietary support or social support. Dietary intervention incorporated personalized dietary advice and nutritional counseling, including motivational interviewing, goal setting, and mindful eating to support adherence to the recommended diet [72]. Social support incorporated a ‘befriending’ protocol following the same visit schedule and length as the dietary support [2]. Results of the study confirmed that the dietary intervention caused a statistically significant decrease of depression symptoms compared to the conventional therapy alone, indicating that an adjuvant dietary intervention, likely involving modulation of the gut microbiome, may be an effective treatment approach for depression.

While two recent RCTs confirmed the benefits of Mediterranean-style diet on depression [1, 3], a diet supplemented with omega-3 fatty acids, selenium, vitamin D, and calcium in the MooDFOOD randomized controlled trial did not reduce major depression episodes in overweight or obese adults with subsyndromal depressive symptoms [73, 74]. However, accounting for only subjects who complied over 12 months, a supplemented diet may be preventive in depression onset.

The PREDIMED randomized trial is the largest dietary intervention study to date, including 7,447 participants between 55 and 80 years old, over a time period of 8 years. The primary endpoint of the study was a reduction of mortality in participants at high risk for cardiovascular disease. The two intervention groups were put on a traditional Mediterranean diet, one supplemented with nuts and the other supplemented with olive oil, whereas the control group was put on a low-fat diet. While the primary endpoint was reached before the study ended, in a secondary analysis of the study results, the researchers assessed whether the dietary intervention decreased the risk for depression. Even though not statistically significant, the average risk for depression for the intervention group was 20% lower. However, in the subset of patients with type-2 diabetes, the risk for depression was 40% lower in the intervention group when compared to the control group, which was found to be statistically significant. In addition to the demonstrated benefit for patients with chronic heart disease, as well as a subset with depression, this study suggests an interconnectedness of two very prevalent chronic diseases: cardiovascular disease and mental illness [3].

The HELFIMED study directly investigated the effects of dietary intervention on self-reported depression. Over a 6-month intervention period, self-reported depressed subjects were put on a Mediterranean-style diet with fish oil and received nutritional coaching and cooking classes for enabling maximal dietary adherence. To account for potential anti-depressive, non-diet related effects of nutritional coaching, the control group received social support. The results of this study indicated a significant reduction in depression, which directly correlated with increased adherence to the Mediterranean diet over 3-months as well as 6-months. Further interesting findings of the study were the association between decreased depressive symptoms with a decreased ratio of omega-6 fatty acids to omega-3 fatty acids [1].

In summary, the benefits of a largely plant-based diet in depression summarized above is likely to include anti-inflammatory effects mediated by increased production of SCFAs and polyphenols, an improvement of intestinal permeability and a resulting reduction in metabolic endotoxemia. Additional benefits from increased consumption of omega-3 fatty acids and trace minerals may relieve the deficiency of certain important nutrients for mood and overall brain health.

### Diet and cognitive decline

Alzheimer’s disease (AD) is a progressive neurodegenerative disease which currently affects over 40 million people worldwide [74]. The disease is characterized by memory loss and a loss of executive function of the brain and can be comorbid with depression, anxiety, and insomnia [75].

Various theories exist about the biochemical mechanisms underlying AD. Biomarkers for AD include the protein aggregates amyloid-beta (Aβ) and tau, which are suggested to undergo disposition in the CNS as part of the pathophysiology [76]. Brainstem regions like the N. solitarius and the locus coeruleus receive vagal input, emphasizing a potential neural link between the gut microbiome and brain areas that are affected by AD as shown in postmortem studies [77]. Neuroinflammation as a precursor of cognitive impairment which also depends on the composition of the gut microbiome, has previously been associated with AD [78]. AD patients have been found to demonstrate decreased levels of systemic primary BAs and enhanced levels of distinct secondary BAs (the product of gut microbial metabolism) when compared to healthy controls, which are directly correlated with impairments in cognitive functioning and brain glucose mechanism [79]. As bile acid synthesis is crucially dependent on dietary factors, and as secondary bile are exclusively generated by certain gut microbes, these findings suggest a possible role of diet and the gut microbiome in the observed alterations in the ratio between primary and secondary BAs. Secondary BA levels were associated with the progression of AD symptoms from mild to severe, with higher levels of secondary BAs being correlated with worse cognitive function [79]. In another study that compared the metabolic profiles of AD, patients to those of healthy controls found the BA deoxycholic acid to be a part of the characteristic metabolites in early diagnosis of AD [80]. A study by Nho et al. found a correlation between specific BAs and changes in brain structure and cerebrospinal fluid (CSF) biomarkers, suggesting that the gut-liver-brain axis plays part in cognitive decline characteristic for AD [81]. All of these data strongly suggest a link between the gut microbiota and the development of AD.

The NUAGE dietary intervention is a one-year trial across 5 European countries investigating the influence of a healthy diet on frailty in pre-frail subjects [6] (Table 1). The premise of this study with 612 participants held that adopting a high-quality diet will modulate the gut microbiome in a way that promotes healthy ageing. Results of this study showed that dietary adherence to a Mediterranean diet was correlated with specific bacterial taxa which were negatively associated with inflammatory markers and also positively associated with markers of low frailty. Furthermore, the researchers performed a microbiome ecosystem network analysis which showed that butyrate producing bacterial taxa (including the taxa *Eubacterium, Feacalibacterium, Blautia and* unclassified *Clostridiales*) which were enriched as a result of the dietary intervention are more central than those taxa that are associated with increased inflammation and reduced cognition, hence frailty.

A higher diet quality in adult life has been associated with a reduced risk of cognitive decline [82]. Moreover, the intake of polyphenols in the elderly has been associated with improved cognitive abilities [83, 84]. Another study showed that a Mediterranean diet supplemented with olive oil and nuts was associated with improved cognitive function in an older population [85]. The observed associations of polyphenol rich diets (which require gut microbes to metabolize into absorbable components) with cognitive function in humans are consistent with earlier preclinical study results [86].

In summary, evidence from preclinical, epidemiological, and a limited number of intervention studies supports the concept that a largely plant-based diet, like the Mediterranean diet is conducive to improved cognition, and a reduction in cognitive decline. These clinical benefits are associated with a reduction in levels of certain secondary BAs, alterations in brain structure, and positive changes in gut microbial composition.

### Ketogenic diet and cognitive decline

Several clinical studies have shown a positive effect of a ketogenic diet on patients with AD or mild cognitive impairment (MCI), consistent with preclinical results [87–91] (Table 1).

Consistent with earlier, preclinical findings [89–94], several clinical studies showed that diets that can induce high blood ketone levels improved cognition and memory in those with AD, with emerging evidence for those with MCI [4, 95–99].

In AD patients or patients with MCI, a ketogenic diet was associated with improvements in cognitive ability as assessed by the Alzheimer’s Disease Assessment Scale – Cognitive Subscale (ADAS-cog) [96], improved verbal memory after 6 weeks on the low carbohydrate diet [95], significantly improved executive function [98] and alterations in relative abundance of gut microbes such as increased *Enterobacteriaceae*, *Akkermansia*, *Slackia*, *Christensenellaceae* and *Erysipelotriaceae* abundance, and decreased *Bifidobacterium* and *Lachnobacterium*, in association with improved AD biomarkers [99]. Several clinical pilot studies additionally confirmed the positive effect of a medium chain triglyceride (MCT) diet on ADAS-cog outcomes in adults with mild-to-moderate AD, with observations of an acute improvement on the ADAS-cog [100], a long-term significant improvement in immediate and delayed logical memory and digit-symbol coding test [4], and an improvement in the ADAS–cog, Memory Composite Score, and episodic memory, language, executive function, and processing speed [97, 101].

In summary, evidence from both preclinical and early-stage clinical studies suggests a therapeutic benefit of a ketogenic diet in improving cognitive function in particular in patients with advanced AD despite the heterogeneity of interventional dietary studies. However, there is a paucity of evidence supporting the effect of a ketogenic diet on the prevention of AD development or in the treatment of MCI, an area of ongoing research.

### Diet and autism spectrum disorder

Autism Spectrum Disorder (ASD) is characterized by impaired social communication and persistent repetitive behavior present in early development and significantly interfering with proper social functioning [102]. Over the past decade and a half, the prevalence of ASD has been increasing dramatically with ASD affecting one in 54 children in the United States today [103]. ASD is often comorbid with gastrointestinal symptoms, anxiety and immune dysregulation [104]. The GI symptoms often associated with ASD include diarrhea, abdominal pain and discomfort, gastric reflux, and alterations in bowel habits [105, 106].

As risk genes predisposing for the development of ASD have not changed during the past decades, various environmental risk factors including diet and environmental toxins have been implicated in its etiology. Several pre-clinical and clinical studies found increased levels of inflammatory markers in the systemic circulation of ASD individuals when compared to neurotypical controls, such as increased IL-1B [107, 108] and heightened systemic TNF-alpha [108–110]. Evidence for increased intestinal permeability has been reported in postmortem analysis of ASD individuals [111]. These observations together with the common comorbidity of GI symptoms and anxiety strongly suggest that gut dysbiosis may be part of the underlying pathophysiology of ASD.

A number of clinical studies [112–115] are consistent with preclinical findings [116–118] in demonstrating altered gut microbial composition and function in ASD patients and suggesting a potential role of the gut microbiome in ASD pathophysiology.

**S**everal small-scale dietary intervention studies have investigated diet as a treatment option for ASD (Table 1). An open-label study in 70 children with ASD investigated the effects of a 12-month dietary intervention consisting of a gluten-free, casein-free diet and found improvement for 81% of the participants after three months [119]. A 12-week, double-blind, cross-over study of a gluten-free, casein-free diet in 14 children with ASD did not report significant differences between the groups but benefits observed by parents which were not identified by the testing were reported [120]. A randomized, placebo-controlled, single-blind study in 54 children investigated how dietary intervention over a 12-month period affects communication, which was evaluated with the Autism Diagnostic Observation Schedule (ADOS). The results reported were significant improvements in communication in the dietary intervention group when compared to the control group. Additionally, the parents who were not blinded for the study confirmed improved social interaction, inattention, and hyperactivity [121]. A more recent randomized, single-blind, controlled study over a 12-month time period involved a comprehensive dietary intervention where a gluten-free, casein-free diet was supplemented with certain key nutrients. The researchers reported significant benefits in nonverbal intellectual ability, greater improvement in ASD symptoms, and developmental age in the dietary intervention group when compared to the control group [7]. Altogether, these studies suggest that ASD core symptoms as well as associated comorbid conditions can be improved through comprehensive dietary treatment interventions, the definite cause of which has yet to be identified and may include but not be limited to changes in the gut microbiome.

Microbial Transfer Therapy (MTT) has emerged as a promising treatment approach for ASD patients, wherein a microbiota transplant from a healthy donor is inserted into the patient. A study with ASD children undergoing MTT found a significant sustained decrease in both GI and ASD symptoms and confirmed favorable changes in the abundance of certain beneficial bacterial taxa, including *Bifidobacteria*, *Prevotella*, and *Desulfovibrio* [113, 122]. These results strongly support the view that the gut microbiome plays a pathophysiological role in ASD. Additionally, this study indirectly suggest that dietary manipulation of the gut microbiome may exert a therapeutic effect mediated by gut microbes. Reported studies about therapeutic benefits of fecal microbial transfer in patient populations for other psychiatric conditions have recently been reviewed with mixed results [123].

In summary, dietary interventions as assessed in these small-scale, poorly controlled clinical studies suggest that a gluten-free, casein-free diet may produce positive effects on symptomatology and severity in some ASD patients, as well as improvements in comorbid GI problems. However, due to the small sample size and the lack of blinding and randomization in most of these studies, dietary interventions in the treatment of ASD remain to be further researched in well-controlled, large-scale trials. Such studies should also evaluate the potential role of diet induced changes in gut microbial function in symptom improvement.

### Ketogenic diet in epilepsy

Although epilepsy is a treatable condition with 1–3% prevalence, 30% of patients continue to have drug‐resistant epilepsy (DRE), or recurrent seizure activity despite taking multiple antiepileptic drugs [124]. The pathophysiology has not been fully elucidated, but intestinal dysbiosis has been implicated, thus making ketogenic diet a potential therapy that can exert antiepileptic effects due to changes in the microbiota. A landmark study in mouse models of epilepsy showed that a ketogenic diet protected against refractory epileptic seizures only in mice that were colonized with certain gut microbiota compared to mice treated with antibiotics [125]. The underlying mechanism involved a change in microbial abundances, leading to a decline in the synthesis of GABA in the periphery while increasing GABA in the CNS to exert antiseizure effects.

Emerging studies in patients with epilepsy have begun to elucidate the change in the gut microbiota after being treated with a ketogenic diet (Table 1). An initial metanalysis of 10 RCTs on the ketogenic diet and drug-resistant epilepsy in children and one RCT in adults found evidence for a small reduction in seizures between treated and untreated groups. However, the study was limited given the rate of attrition and short follow-up time frame of 6 months or less [126].

Some studies have evaluated the effect of a ketogenic diet on gut microbial composition. In six patients with DRE and glucose transporter type 1 deficiency syndrome who followed the ketogenic diet for three months, reverse transcription polymerase chain reaction analysis of the fecal microbial profiles showed no change in *Firmicutes* or *Bacteroides* while showing a significant increase in *Desulfovibrio spp*, which is involved in gut mucosal inflammation [127]. However, in a more recent study, shotgun metagenomic DNA sequencing was applied to fecal samples from twelve children with DRE before and after three months on the ketogenic diet. While taxonomic and functional profiles were altered, alpha diversity did not change significantly during the dietary intervention. Relative abundance of the butyrate producing taxa *bifidobacteria*, *E. rectale*, and *Dialister* were significantly diminished during the intervention, while *E. coli* was increased [128]. A case‐control study on 14 children with DRE and 30 healthy controls showed that a ketogenic diet for a week led to a 50% reduction in seizure frequency in infants, associated with decreased levels of *Proteobacteri* (*Cronobacter*) and increased levels of beneficial *Bacteroidetes* taxa (*Bifidobacterium*, *Bacteroides*, and *Prevotella*) compared to baseline, correlating with suppression of seizure activity [108]. A study of 20 children treated with a ketogenic diet for six months showed different patterns of changes in microbiota abundance, with generally lower levels of *Firmicutes* and *Actinobacteria* and increased levels of *Bacteroidetes* [129]. There was also heterogenous patient outcomes, where a subgroup with increased abundance of butyrate producers (*Alistipes*, *Clostridiales*, *Lachnospiraceae*, *Ruminococcaceae*, and *Rikenellaceae*) had a less than 50% reduction in seizures compared to other subgroups.

When viewed together, there is emerging but mixed clinical evidence tying the ketogenic diet to changes in the gut microbiota and improvements in seizure activity in subsets of patients. Given the dysbiosis that is a consequence of the ketogenic diet, empirical trials of pre- or probiotics may be considered in conjunction with a ketogenic diet in children with DRE, and longer-term RCTs are needed to assess the feasibility, including side effects.

### Challenges

Published studies collectively show possible therapeutic benefits from nutritional interventions in chronic brain disorders, an important step in an area which has not seen major advances in the development of novel medications [130]. However, several challenges remain before the role of diet in the treatment of depression, anxiety, cognitive decline, epilepsy, AD and ASD is firmly established (summarized in Table 2). While a growing list of findings observed in animal models of these diseases strongly suggest a causative role of the gut microbiome and indirectly diet in behavioral and biological alterations, the translatability of preclinical findings into human psychiatric diseases has been challenging in part due to heterogeneity of human study populations, interindividual differences in genetic vulnerability, confounding environmental factors and considerable differences in structure and function between the human and mouse brain. In addition to the challenges to clearly identify a causative role of the gut microbiome, high quality reports about diet-induced normalization of disease-associated dysbiosis which result in behavioral improvements are largely missing. Detailed and standardized characterization of the gut microbial composition down to the strain level, and functional characterization using shotgun metagenomics, meta-transcriptomics and metabolomics has only been used in a minority of recent studies. Information about the benefits of certain diets largely comes from cross sectional, epidemiological studies. These studies have primarily shown a benefit of a largely plant based diet in slowing cognitive decline [6, 85], or in being associated with a lower rate of depression [1–3]. However, there is a paucity of high quality, randomized controlled clinical trials, and with the exception of a few studies in depression, cognitive decline and in intractable epilepsy, there is relatively little evidence to date that such dietary interventions show a significant therapeutic benefit. The implementation and control of a standardized diet over long periods of time is challenging. The assessment of dietary habits in most studies has relied on questionnaire data which have been notoriously unreliable. Furthermore, in the reviewed publications, the way diet was measured using different questionnaires and interviews varied greatly between each study, leaving many variables unaccounted for. Future studies may use metabolomics (“Fodomics”) to objectively determine the dietary habits of study participants [131, 132].

**Table 2:** Challenges in Nutritional Psychiatry.

Challenges
Poor translatability of preclinical findings into human psychiatric diseases due to heterogeneity of human study populations, confounding environmental factors and significant interspecies differences in brain structure and function.
Paucity of high quality RCT showing diet-induced normalization of disease-associated dysbiosis causally related to clinical improvements.
Detailed and standardized characterization of the gut microbiome down to the strain level, and functional characterization using shotgun metagenomics, meta-transcriptomics and metabolomics has only been used in a minority of recent studies.
Methodological limitations in assessing dietary habits using questionnaires and interviews
Implementation of and adherence to a standardized diet over long periods of time has been challenging.

A major unresolved question is the disease specificity of altered gut microbial signaling mechanisms. As discussed in this review, there appears to be a limited number of such mechanisms, which have been identified in different brain disorders, including but not limited to immune signals, anti-inflammatory mechanisms (SCFAs), tryptophan metabolites, and secondary bile acids. Even though comprehensive assessment of gut microbial functions is not available for all disorders to date, it is plausible that a relatively limited number of such signaling mechanisms in genetically vulnerable individuals plays a role in such different disorders as ASD, depression, AD and MDD. Consistent with this hypothesis are reported findings from the American Gut Project showing that individuals who reported a mental disorder such as depression, schizophrenia, post-traumatic stress disorder (PTSD) or bipolar disorder had more in common with other people with mental disorders, in terms of the bacteria makeup of their gut microbiomes, than they did with their mentally healthy pairs. This observation held true in both U.S. and UK populations as well as in males and females [133].

### Clinical implications and future directions

Until an objective therapeutic benefit of specific dietary interventions has clearly been established, practical implications are largely limited to the general recommendation of a healthy, largely plant-based diet similar to the traditional Mediterranean diet which has clearly been shown to be associated with an increased abundance of diverse and rich gut microbiome species with a high abundance of anti-inflammatory SCFA producers, including *F. prausnitzii*, *E. rectale*, *Roseburia* and *A. mucinophilia* (Table 3). As discussed extensively in this review, systemic low grade immune activation due to increased gut permeability and reduced abundances of SCFA producers appears to be a shared feature of several common chronic brain disorders and increasing the prevalence of butyrate producing microbes in the gut should be a general therapeutic strategy. High quality RCT demonstrating benefits beyond adherence to such a “microbiome-friendly” diet by the intake of supplements in the form of pre, pro- or postbiotics (substances produced through and released by the metabolic activity of the microbiome) are currently not available, even though they may have small additive therapeutic effects [134]. For example, the intake of a consortium of butyrate producers may improve metabolic abnormalities and reduce systemic immune activation [135]. The evaluation of the potential therapeutic benefit of fecal microbial transplants in brain disorders, mimicking such effects previously reported in animal models has shown inconsistent and time limited success [123], with the exception of the study in ASD discussed earlier. However, confirmation of these findings in a RCT is essential before recommending it as an effective treatment. Diagnostic testing of the gut microbiome to develop personalized dietary approaches, or to determine the future risk for the development of chronic brain disorders is in its early stages, and the benefits of such testing have not been demonstrated in prospective, longitudinal studies. The usefulness of basing personalized, specific dietary and supplement recommendations on stool microbiome assessments is awaiting objective evidence, even though such testing, together with assessment of genetic risk factors may become a useful approach in the future. For now, personalization of a largely plant based diet to avoid gastrointestinal side effects in particular bloating, gas and abdominal discomfort in patients with psychiatric diagnoses and often comorbid gastrointestinal symptoms, is best done empirically. Patients should be started on a Mediterranean-like diet and encouraged to carefully identify food items that reproducibly generate symptoms. Elimination of a small number of such foods will enable the patient to avoid GI symptoms, while eating the optimal, personalized diet for brain health. Despite the limitation of currently available information, there are several recommendations that are supported by a body of preclinical and clinical evidence, including the adherence to an anti-inflammatory, Mediterranean type diet, and the inclusion of dietary counseling in addition to pharmacological and behavioral multidisciplinary treatment strategies.

**Table 3:** Future directions in Nutritional Psychiatry.

Clinical implications/future directions
Until an objective’s therapeutic benefits of specific dietary interventions have clearly been established, effective treatments are limited to the general recommendation of a healthy, largely plant-based diet with high variety of fruit and vegetables, or a ketogenic diet in some instances.
Results from high quality RCTs demonstrating benefits beyond adherence to a largely plant based (“microbiome-friendly”) diet by the intake of supplementary pre-, pro- or postbiotics are currently not available.
Potential therapeutic benefits of fecal microbial transplants in brain disorders, mimicking such effects previously reported in animal models, and in human C difficile colitis has generally not been successful, with the exception of an uncontrolled study in ASD (Kang et al., 2017; Kang et al; 2019). Confirmation of these findings in an RCT is essential before recommending in as an effective treatment for ASD. Reported studies about therapeutic benefits of fecal microbial transfer in patient populations for other psychiatric conditions have recently been reviewed with mixed results (Chinna Meyyappan et al., 2020).

One of the major challenges of Nutritional Psychiatry Research is to gradually change the prevalent mindset shared by a majority of researchers and practitioners that psychiatric disorders are diseases of the brain, and do not involve the gut and its microbiome. An education of mental health professionals about the crucial role of diet and its effect on brain gut microbiome interactions, and the need for and interdisciplinary approach to this field is necessary to make progress in this area of psychiatry [136].
